# Ending tuberculosis: challenges and opportunities

**DOI:** 10.3389/ftubr.2024.1487518

**Published:** 2024-12-24

**Authors:** Beth Gilmour, Kefyalew Addis Alene

**Affiliations:** ^1^School of Population Health, Faculty of Health Sciences, Curtin University, Perth, WA, Australia; ^2^Geospatial and Tuberculosis Team, The Kids Research Institute, Perth, WA, Australia

**Keywords:** tuberculosis, challenges, opportunities, elimination, SDG

## Abstract

Despite impacting mankind since ancient times, tuberculosis (TB) persists as the leading cause of death from an infectious disease. TB can remain latent and further research is required to understand activation risk and the risks vs. the benefits of treating latent infection. Drug resistance poses an escalating threat to treating active disease and achieving cure. Recent advances in molecular and epidemiological techniques facilitate early diagnosis, drug susceptibility testing and an opportunity to better understand transmission dynamics. Research is ongoing to develop safe, efficacious tolerable drug regimens and the challenges of antibiotic resistance have led to a resurgent interest in therapeutic alternatives. Vaccine development is challenged by the pathogen's genetic diversity, the heterogeneity of host susceptibility and the extreme complexities that occur across the interactions between TB and its host. Across all stages of TB pathogenesis, developments in artificial intelligence, geographic information systems, digital health technologies, renewable energy solutions and nano medicine are providing opportunities to improve TB control. Resource constraints however often challenge the opportunity to access these new technologies by those most in need. The societal inequalities in accessing new technologies further compound socio-economic and health related TB determinants Addressing these complex determinants which include malnutrition, HIV infection, diabetes, substance abuse, poor environmental conditions and multi-factorial barriers to health care access, will require political will, sufficient funding, and a holistic multisectoral response.

## Introduction

With respect to mortality and morbidity, tuberculosis (TB) is one of the most important infectious diseases that has affected mankind since ancient times. Although the genus *Mycobacterium* is hypothesized to have originated more than 150 million years ago (1), the disease still continues to take a huge toll on society and in 2023 was estimated to have resulted in the loss of 1.25 million lives (2). In 2019, 66 million disability life years (DALYs) were attributed to TB (3), but due to the exclusion of post-TB sequelae in the DALY calculation, the true burden of morbidity is likely to be significantly higher (4). The pathogen's ability to evade the hosts immune response and elicit effective transmission, and the inequitable nature of mankind have contributed to the disease eluding eradication (5, 6).

The *Mycobacterium tuberculosis* complex (MTBC) contains a group of genetically similar bacteria capable of causing TB across a range of mammalian hosts (7). *M. tuberculosis* (Mtb) is the main etiological agent of TB in humans, and *M. africanum* is also prevalent in Africa, with each of these species having different lineages that have distinct characteristics in terms of pathogenicity, transmissibility, drug resistance and geographic origin (8, 9).

Mtb is an obligate intracellular pathogen and although it has been established that transmission occurs by bioaerosol, the complexities of transmission are not fully understood and are considered a research priority (10). Mtb has evolved to infect, survive and replicate within human phagocytic cells and although the lungs are the primary site of infection, the bacillus are able to infect any organ in the body via hematogenous routes (10). The intracellular nature of the pathogen facilitates long-term latency which translates to active disease when host immunity is compromised (11). Host immunity is highly heterogenous and the spectrum of disease is hard to differentiate and identify, resulting in fundamental gaps in our knowledge of its features and implications (10, 12). The ability to correlate the spectrum of subclinical disease with symptoms, clinical signs and diagnostic tests is complicated in endemic settings, where exposure is difficult to identify and often multitudinous (12). Mtb encounters a range of host environments at different stages of infection and although our understanding of the host/pathogen determinants and mechanisms that promote a successful infection are poorly understood, the pathogens unique and dynamic cell envelope is thought to play a key role (13). The cell envelope is the target of most current and proposed therapies, but more work needs to be undertaken to understand the cell envelope composition across Mtb strains with different antibiotic resistance profiles (14). Antibiotic resistance is conferred by genomic mutations and propagated through replication of resistant Mtb and onward transmission (15). Mtb has acquired resistance to all the antibiotic drugs that have been used against it and there are concerns that current targeted genetic diagnostics could create their own selective pressures (15).

Although globally ubiquitous, the prevalence of infection shows wide variation by region and social context. TB is associated with low socio-economic status and is a disease that fuels the cycle of poverty with more than 80% of cases and deaths occurring in low- and middle-income countries (6). In addition to socio-economic development, TB is influenced by health-related risk factors including HIV infection, malnutrition, diabetes and drug and alcohol abuse (16). Although all age groups are at risk of infection, the disease has its greatest impact on adults in their productive years (6). In 2022, the greatest number of new cases occurred in the World Health Organization (WHO) South-East Asia Region (46%) followed by the African Region (23%), with 87% of cases occurring in 30 high TB burden countries and more than two thirds of the global total befalling eight countries (6).

Within the targets of the 2030 Sustainable Development Agenda, adopted by members of the United Nations (UN) in 2015, was a commitment to end the TB epidemic by 2030 (17). The WHO End TB Strategy builds upon and expands the scope of efforts in the context of the UN Sustainable Development Goals (SDG). The End TB Strategy has targets of a 90% reduction in TB deaths and 80% reduction in TB incidence by 2030, relative to a 2015 baseline (18). Progress toward the interim targets for the 2030 goals, is severely off track with 2015–2021 net reductions in TB incidence and death only one fifth and one-tenth of the way to the 2025 milestones respectively (19). The 2018–2022 WHO status report on TB treatment, preventative therapy and funding, also show progress to be significantly off-track (20). Examples of the shortfalls show only 19% of targeted children with multi-drug resistant TB (MDR-TB) received treatment, only 10% of targeted household contacts aged < 5 years received TB preventative treatment, and that funding for TB services only reached 44% of that targeted in 2022 (20). Modeling shows that if the 2030 mortality target is not achieved until 2045, there will be the loss of an additional 5.7 million lives and an estimated economic cost of $3.0 trillion (21).

If we are to achieve the End TB Strategy targets a holistic, multisectoral response will be required that optimizes current control program strategies, addresses social determinants, eliminates inequities and that invests in research and development (22) This review seeks to discuss the challenges and opportunities in achieving these targets and is structured to consider aspects relevant to the three End TB Strategy pillars. Pillar I: “integrated, patient-centered care and prevention,” pillar II: “bold policies and supportive systems” and pillar III: “intensified research and innovation” (22).

## End TB strategy pillar I: integrated, patient-centered care and prevention

### TB diagnosis and treatment

Efforts to combat the TB pandemic have focused on early diagnosis, appropriate treatment, active case finding and the administration of preventative therapy to people with latent infection who are at high risk of developing active disease (23). Delivering these strategies will require tools capable of detecting latent TB infection (LTBI), the ability to predict the risk of latent infection progressing to active disease, the capability to detect active disease and any drug resistance and the administration of the correct treatment regime (24).

The diagnosis of LTBI is indirect and reliant upon the measurement of an immune response to Mtb antigens (25). Currently there are no diagnostics available to differentiate between an immune response due to LTBI vs. active disease and the distinction is reliant upon clinical, bacteriological and radiological findings (25). Methods used to identify LTBI include the tuberculin skin test (TST), *M. tuberculosis* antigen skin test (TBST) and interferon gamma release assays (IGRAs) (25, 26). There is no recommended gold standard for LTBI testing and each of the available methods have their challenges, including the requirement for multiple appointments and a cold chain for the skin tests and cost and time sensitivity for the IGRAs (26). The global prevalence of LTBI is unknown but estimates of 25% have been made (27). Literature quotes the lifetime risk of LTBI developing into active disease to average 5%−10% but figures are based upon a single dated study (28), highlighting the need for comprehensive up-to-date research on the activation risk. Latency is not stable and bacterial replication can be intermittent, transient or progressive leading to incipient and subclinical infection prior to active disease (25). A number of approaches to estimating activation risk are being investigated including the use of gene signatures and transcriptional biomarkers, but further research is required (29, 30). The prophylactic treatment of patients with LTBI at risk of progressing to active disease is a key component of the End TB Strategy, but this needs balancing against the potential adverse effects of treatment (31). LTBI therapy is challenged by sub-optimal treatment adherence rates, the absence of a regime to treat latent MDR strains, and resource deficits as many countries prioritize programs that address active disease (32, 33). Amongst the key “at risk” populations prioritized for LTBI testing and treatment are people living with human immunodeficiency virus (PLHIV) and household contacts of bacteriologically confirmed TB cases (31) Additional high risk population groups include healthcare workers, immigrants from high TB burden countries, the homeless and illicit drug users (34). The diagnostic methods used to identify a ‘bacteriologically confirmed' case of TB include culture, sputum smear microscopy, molecular testing and lateral flow urine lipoarabinomannan (LF-LAM) assays (35). Bacteriological confirmation is required to facilitate drug susceptibility testing and in the absence of this confirmation, the case is classified as a clinical diagnosis (i.e., based on clinical symptoms, chest radiograph abnormalities) (35, 36). Culture is the gold standard diagnostic test with the capacity to detect drug resistance and new mutations, but it has the disadvantage of a long mean time to detection and for many resource limited settings sub-optimal laboratory facilities restrict its utility (37, 38). For many countries, culture is reserved for cases of treatment relapse (38). Microscopy is the primary method of diagnosis in resource constrained environments and although less sensitive than culture, it has the advantage of simplicity, reduced cost and rapidity (39). Although advances have been made in improving sensitivity (e.g., from traditional Ziehl-Neelsen staining to light-emitting diode microscopy) efficacy is operator dependant and the need for a large bacillary load limits its application for PLHIV, extrapulmonary and childhood diagnoses (40, 41). Microscopy also has the disadvantage of the inability to differentiate between MTBC and nontuberculous mycobacterium (NTB) and between live and dead bacilli (38, 42). Recent advances in molecular diagnostics include nucleic acid amplification tests (NAATs) which facilitate an early diagnosis of disease with high sensitivity and specificity (43). The WHO now recommend that rapid techniques be used as the initial diagnostic (44). NAATs have the advantage of detecting resistance to selected anti-TB drugs, the ability to test at point-of-care with a rapid turn-around and although they are not capable of differentiating between live and dead bacilli they have increased accuracy vs. microscopy especially in patients with paucibacillary disease and PLHIV (43–46). These molecular techniques are also able to utilize stool as an alternative specimen diagnostic which provides an opportunity to address the case detection gap in children, which is greatest in those under 5 years of age (47). Despite the advantages offered by NAATs, there are challenges to accessing these technologies in resource constrained environments including cost, complexity, the need for power, and reagent supply chain and storage considerations (37, 48). Another recent biomolecular development is the lateral flow urine lipoarabinomannan assay (LF-LAM) which detects lipopolysaccharide in the wall of metabolically active or degenerating mycobacterium cells (37, 49). LF-LAM has suboptimal sensitivity for general screening but has improved sensitivity in patients with human immunodeficiency virus (HIV)-TB coinfection (49). LF-LAM sensitivity has been shown to increase with decreasing CD4 cell counts and this method also has utility where sputum samples cannot be obtained (49). Another TB screening test of utility for PLHIV is the immunological marker C-reactive protein, especially in situations where other recommended screening tools such as chest X-ray are not readily available (44, 50).

Line probe assays (LPA) are molecular assays that can be used on smear-positive and culture isolates following DNA extraction and amplification to differentiate genetic mutations associated with drug resistance (46). In addition to the identification of drug resistance, LPAs can facilitate the identification of pre-extensively drug resistant (XDR) and XDR-TB in MDR-TB isolates but they are complex to perform and the geographical variation in mutation prevalence requires regional level sensitivity and specificity baseline assessment (46). Whole genome sequencing (WGS) provides an opportunity for genotypic drug susceptibility testing (DST), bacterial strain identification and the ability to differentiate relapse from re-infection thereby facilitating a better understanding of transmission dynamics which can inform improved contact tracing (51). Although these new technologies have numerous advantages, resource constraints make their extension to many jurisdictions prohibitive and there is a need to address financial constraints and implementation gaps (52).

In combating active disease, technological capability is only one part of the jigsaw and timely diagnosis is vital to interrupting transmission and optimizing treatment outcomes (53). There are an estimated three to four million active TB cases per year that are undiagnosed, with 75% of these cases occurring in 13 countries (54). To identify missing cases, the WHO recommend active case finding (ACF) in high risk populations (55). High risk populations include contacts of index cases and PLHIV, with other at-risk groups being situation specific depending upon the epidemiological, social and health system context (55). ACF interventions are context specific and are influenced by resource availability, physical geography and health system capability (56). For many low- and middle-income countries, resources limit the capacity to undertake ACF and diagnosis is dependent upon passive case finding (PCF) (57). For both ACF and PCF there are patient related (e.g., low TB literacy and education, cost- indirect and direct expenses, stigma and anxiety) and healthcare related (e.g., capacity, capability and resource) barriers to optimizing diagnosis (58). The barriers to patient health seeking behavior are complex and setting specific necessitating the need for local solutions (59). The interface between patient and healthcare barriers are also setting specific and often complicated by the mix of traditional, private, and public providers (59). Although global initiatives have been implemented [e.g., Global Laboratory Initiative (GLI)] to expand and accelerate access to quality assured laboratory services (60), provider specific delays (e.g., scarcity of healthcare professionals and infrastructure, low index of suspicion for TB and weak referral links) (61, 62) need addressing at a local level.

To be successful, effective diagnosis must be paired with correct and timely treatment and research is ongoing to develop safe, efficacious tolerable drug regimens capable of eradicating the disease and preventing long-term sequelae associated with the disease or resulting as an adverse effect of treatment (37). For both drug-susceptible TB (DS-TB) and drug-resistant TB (DR-TB), significant advances have been made in optimizing and re-purposing, identifying new compounds, and shortening treatment durations without adverse effect upon efficacy (63). In addition to efficacy, is the need to improve patient adherence and in parallel with shortened regimen durations, alternate routes of drug delivery are being evaluated (37). At August 2023 there were 28 TB drugs in the clinical trial pipeline, of which 18 are new chemical entities, seven are repurposed, two drugs (bedaquiline and delamanid) have received accelerated regulatory approval and one drug-pretomanid has received approval by the United States (US) Food and Drug Administration (64). Following these drug development advances, the WHO has endorsed new TB treatment regimen recommendations. In addition to the existing recommended regimen of 6 months of rifampicin, a new shorter 4-month course of isoniazid, rifapentine, moxifloxacin and pyrazinamide has been endorsed for DS-TB (65). Ethionamide has also been included in the essential medicine list for the treatment of DS-TB (66). Although the new regime has the advantage of a shorter treatment duration, it has the disadvantage of an increased pill burden and a substantially higher cost, due primarily to the inclusion of rifapentine (65). It is hoped that further developments will lead to reduced costs and a fixed dose combination tablet (65). For patients with MDR/rifampicin resistant (RR) TB a new all oral, shorter 6 month regimen of bedaquiline (B), pretomanid (Pa), linezolid (L) and moxifloxacin (M) collectively referred to as BPaLM has been endorsed (67). For patients with pre-XDR-TB the regimen can be applied without moxifloxacin (BPaL) (67). Although these new regimens offer benefit, they are complex and require clinical management especially in patients with comorbidities (68). New treatment regimens will require long-term appraisal for safety, tolerability, efficacy, and disease relapse rates, plus ongoing evaluation when administered with concurrent antiretroviral therapy (ART) (24). For these new regimens, further work is also required to improve genotypic-phenotypic concordance limits to facilitate the development of molecular DST (24).

Although the WHO have endorsed a number of new treatment regimens (66), certain population groups (e.g., children, PLHIV with a CD4 count < 100 cells/mm^3^, pregnant, breastfeeding and post-partum women) are excluded due to a lack of safety data, necessitating the need for further research (65, 68). Another limitation of the new recommendations is the lack of consideration for disease severity which can translate into undertreatment for patients with severe disease, and unnecessary treatment with potential toxic side effects for patients with a lower disease burden (69). Research is ongoing to identify methods to predict treatment response and the likelihood of relapse, so drug combinations and durations can be tailored with greater specificity for each patient (69).

The duration, complexity and side effects of treatment create a challenge to adherence. Poor treatment adherence leads to prolonged infectiousness, poor treatment outcomes and the development of drug resistance (70). Interventions that have been reported to be effective at increasing adherence include direct observation therapy (DOT), text reminders and education, drug box reminders, counseling and voucher interventions (71). Although interventions like DOT have been widely adopted, there is controversy regarding its reported effectiveness (70), which highlights the complex and dynamic nature of treatment taking behavior which in itself may change over time (72). Community involvement throughout the TB care cascade has been shown to be valuable in addressing communication and implementation gaps that often relate to complex cultural, social and economic interrelations (73).

### Drug resistant TB

Antimicrobial resistance (AMR) is one of the greatest threats to public health and development and one that will further exacerbate inequalities (74). Modeling shows AMR will result in an annual global economic shortfall of $1–$3.3 trillion by 2050 (75). DR-TB accounts for a disproportionately large amount of the global AMR burden and if left unchecked threatens to reduce the world economy by 0.63 per cent annually (76, 77). The extent of TB drug resistance- from mono (primarily isoniazid and rifampicin)- to extensive (isoniazid and rifampicin, a fluoroquinolone, and a second-line injectable or bedaquiline or linezolid) has been increasing over time (78, 79). The escalation in resistance brings an escalation in the challenges to achieving cure. Combating DR-TB requires more protracted treatment with increasingly complex, toxic, and costly regimens (80). In 2020 the direct cost of treating DS-TB in the U.S. was estimated to average $20,000/patient, a figure that escalates to $568,000 for extensively resistant TB, estimates that would be significantly higher if the indirect costs borne by patients were also considered (78).

DR-TB can arise through intra-patient evolution of resistance (acquired resistance) or through the direct transmission of genetically resistant bacteria (transmitted resistance) (76). Acquired resistance develops due to inadequate treatment and can be further amplified by the repeated use of short-course chemotherapy (81). A “One Health” approach to tackling drug resistance acknowledges the complexities of the issue and also provides an opportunity to consider the reservoir of infection and consequence of TB infection in other host species (82). Advances in molecular and phenotypic methods have revolutionized the diagnosis of drug resistance, but access to DST is still poor in many parts of the world (81). Universal DST is a key component of the End TB Strategy (18), and is fundamental to halting escalating resistance and retaining drug efficacy (83). To implement DST, policies within and between countries will need aligning and funding restraints and healthcare limitations will need addressing (84). To limit DR-TB transmission, active case finding, rapid diagnosis (including DST) and prompt effective treatment are key (76). Active case finding can employ a range of strategies from education campaigns and community mobilization through to systematic screening depending on resource availability (56). The WHO have recently recommended novel DR-TB regimes which have shorter treatment durations, lower toxicity and improved efficacy and should therefore improve treatment adherence and as a result the development of further resistance and continued transmission (68). Although these new treatment regimens offer significant benefits for patients and cost savings for their respective governments (85), more needs to be done to ensure pharmaceutical companies facilitate generic competition equitably and that they do not continue to price gouge (86).

### Zoonotic TB

Although a number of species within the MTBC have been demonstrated to cause zoonotic TB (zTB) including *M. canetti, M. bovis, M. caprae, M. microti, M. pinnipedii, M. mungi*, and *M. orygis*, current zTB estimates are solely based on *M. bovis* (87). In addition to the exclusion of many species, zTB estimates are challenged by the inability to clinically differentiate between *M. bovis* and *M. tuberculosis* infection and a lack of surveillance data in both human and animal populations, especially in countries where bovine TB is endemic and laboratory capacity is limited (88). Although the true burden of zTB is unknown, an estimated 140,000 cases (range 69,800–235,000) occurred globally in 2019 (89). The detrimental impact of infection goes beyond human health, with impacts on other species, livelihoods, and the conservation of wildlife (88). In addition to the large reservoir of infection across host species, is the challenge of reverse zoonoses with the concern that this includes the bi-directional transmission risk of DR-TB (87). *M. bovis* is naturally resistant to pyrazinamide and some isolates have also been reported as resistant to rifampicin and isoniazid (88).

The Stop TB Partnerships Global Plan to End TB 2016–2020 now identifies people in contact with livestock as a key at TB risk population and a “One Health” approach is being advocated which recognizes the interdependence of the health of people, livestock and the environment (87, 88). Recommendations to address zTB include expansion of appropriate diagnostic tools and capacity to identify zTB in people; development of appropriate sample collection methods (as many cases of zTB are extrapulmonary); expanded coverage of DST; improved diagnosis, surveillance, control and elimination programs in livestock and the consideration of wildlife as a reservoir of infection; improved food safety standards; improved surveillance and reporting; improved intersectoral and collaborative approaches to developing and implementing policy (88). Implementing these recommendation will require political will and adequate funding (88).

### TB vaccines

Vaccines have the potential to significantly reduce TB incidence, mortality, and societal costs whilst also mitigating AMR (90). At present however there is only one vaccine- Bacillus Calmette–Guérin (BCG)-that is licensed to combat TB (91). BGC was deployed in 1921 and despite its administration to more people in the world than any other vaccine, controversy exists regarding its efficacy (91–94). The vaccine provides protection against tuberculous meningitis and miliary TB in infants and young children and is usually administered as part of the routine newborn immunization schedule (95–97). In adults, the effectiveness of the vaccine against both the acquisition of infection and the development of active pulmonary disease, varies widely across studies (92, 96, 98, 99).

In the quest for a BCG replacement, three vaccination strategies have been proposed: one that prevents primary infection and disease progression following exposure; one that prevents the development of active disease from LTBI and an immunotherapeutic adjunct to accompany TB treatment in patients with active disease (100). To guide vaccine development the WHO has documented the preferred product characteristics for therapeutic and prophylactic vaccines (101, 102), and as of August 2023, there were 16 vaccine candidates in the clinical development pipeline (64). There are currently four novel vaccine candidates in phase III trials (64), including GamTBvac a recombinant subunit BGC booster vaccine aimed at preventing pulmonary TB in HIV negative adults aged 18–45 years (103). *Mycobacterium indicus pranii* (*Mycobacterium w*) (MIP) vaccine (also known as Immuvac), an immunotherapeutic and immunoprophylactic agent approved to treat leprosy, is also being evaluated for its utility in the treatment of TB (104). MTBVAC is the only vaccine candidate in phase III trials which is based upon an attenuated human clinical isolate of *M. tuberculosis* (105). MTBVAC aims to replace BCG as a preventative vaccine for newborns and also serve as a booster for adults and adolescents (106). The fourth phase III candidate is VPM1002, a recombinant BCG (rBCG) vaccine that is being evaluated for its efficacy in preventing infection in newborns and the reoccurrence of disease in individuals who have been successfully treated (107–109).

Vaccine efficacy is impacted by the extreme complexities that occur across the interactions between MTB and its host (109). Pathogen virulence and host immunity, and the interactions between these impact infection prevalence, disease progression and TB outcome (109). Although there have been significant advances in molecular biology and bioinformatics (110, 111), one of the greatest challenges is the lack of biomarkers which signal the prospective risk of developing disease or the correlates of protection (90). TB vaccine development is also limited by the lack of a suitable animal evaluation model (106). Findings from “successful” animal studies do not always extrapolate, as evidenced by the vaccine candidates that are terminated once they enter human trials (112). It is hoped that novel humanized transgenic animal models will play a future role in pre-clinical evaluations and that new technologies will facilitate new opportunities within existing models (112). Another challenge is the lack of emulated exposure variables- those employed in animal models (typically high dose, single exposure, single strain) differ from those experienced by the human host in natural conditions (typically low dose, multiple exposure, multiple strains) (106, 113). The discord between animal models, and clinical trial endpoint criteria, adds another complexity to candidate vaccine evaluation (106). In preclinical trials, efficacy is assessed in terms of improved bacterial counts, reduced histopathological damage and increased longevity, whereas TB incidence is used to determine vaccine effectiveness in clinical trials (106).

Vaccine development is also challenged by the complex and diverse heterogeneities of TB epidemiology. In addition to pathogen genetic diversity, and the heterogeneity of host susceptibility to infection, is the large variation in confounding variables between and within populations e.g., nutritional status, socio-environment determinants, co-infection/co-morbidities etc. (114). The infection background of a population is also heterogenous, and complicated by BCG vaccination and nontuberculous mycobacteria (NTM) infections (106).

Despite these challenges, the delivery of an effective vaccine for SARS-CoV-2 within 12 months of its discovery, demonstrates what can be achieved when there is collective political will and sufficient funding (115). Whilst genomic and pathogenicity differences between MTB and SARS-CoV-2 are acknowledged, would a vaccine solution be fast tracked if TB posed an imminent threat to the developed world? (116). Despite modeling studies showing novel TB vaccines to be impactful and cost effective in low- and middle-income countries, and investment cases demonstrating clear and significant returns on investment, there is a lack of political and funding momentum (90, 116, 117). To address this lack of impetus, the WHO has established a TB Vaccine Accelerator Council which aims to replicate the successes achieved in the SARS-CoV2 vaccination program whilst also acknowledging lessons learnt and the need to ensure vaccine equity (91, 118).

## End TB strategy pillar II: bold policies and supportive systems

### Social determinants of TB

Social determinants of health (SDH) are the non-medical factors that influence health outcomes and include social and economic policy, social norms, development agendas, political systems and climate change (119). SDH are estimated to account for 30%−55% of health outcomes which highlights the need to employ a holistic approach to disease eradication (120, 121). SDH are drivers of health inequity and must to be addressed if global targets for TB eradication are to be achieved (16, 121). Communicable disease programs have made significant progress when SDH are addressed, as has been demonstrated for HIV/AIDS (120).

Social determinants are of significance across all stages of TB pathogenesis and impact exposure to infection, disease transmission and progression, timely and accurate diagnosis, treatment adherence and treatment outcomes (120). The population distribution of TB mirrors the distribution of these social determinants and highlights the inequities between and within societies (120, 122). For some populations e.g., migrants and refugees, the social determinants of TB are compounded, thereby exacerbating their vulnerability to disease and impacting their ability to access appropriate treatment (123, 124).

For TB, poverty is often the underlying cause of numerous risk factors including malnutrition, poor living and environmental conditions and is associated with poor health knowledge, limited access to healthcare, and a lack of empowerment (125). The lack of education and empowerment then leads to further exposure to additional risk factors such as HIV, drug, and alcohol abuse (125). It can be argued that poverty is a policy decision and that inequities are effectively established by the government of the day at levels they are happy to contend with (126, 127).

Addressing the complexities of poverty will require international commitment to short and long-term solutions (128). Although food assistance is critical to preventing malnutrition, it is only an interim solution and long-term investments in sustainable agriculture are required (129). Social protection and microfinance initiatives, offer an opportunity to provide short-term assistance whilst providing an opportunity for long-term human capital development (120). Universal health coverage (UHC) is considered a key component to achieving the End TB Strategy and is defined as universal access to healthcare without incurring financial hardship (130, 131). Although this is a recommended component of the End TB Strategy and improvements in UHC have been shown to be effective in improving other health outcomes e.g. child mortality (132), further research is required to understand its causal effect on TB morbidity and mortality (133). Over the last decade there have been improvements in health service coverage globally, but at the same time there has been an increasing trend in the number of people facing catastrophic expenditure on health (134). Barriers to accessing TB healthcare are not however solely economic, and geographic, social, cultural and health system barriers must also be overcome (135). Socio-cultural barriers include inadequate knowledge about TB, belief systems and traditional medicine, stigma, and cultural norms that result in gender based imbalances in accessing care (136). Education and the cultural competence of health care providers are key to addressing these socio-cultural barriers (137, 138). Health system barriers often reflect organizational capacity constraints in terms of infrastructure, resources, co-ordination, and workforce limitations, the latter of which is also impacted by stigma (139–141). However, an overarching barrier to all aspects of healthcare delivery is corruption, to which $560 billion per year is estimated to be lost (142, 143). Education, in conjunction with policies that reduce supply and demand, will play an important role in addressing behavioral TB risk factors such as drug and alcohol abuse (144). Implementing control policies in developing countries can however create an economic conflict, due to the significant value of for example tobacco exports (144). A possible solution to overcoming this conflict is to integrate tobacco cessation interventions into TB control programs (145). Vertically integrated TB control programs also provide an opportunity to address the latent synergies of HIV and diabetes (146).

The inequities that impact TB have been further exacerbated by the recent COVID-19 pandemic, conflict, and climate change (129, 147). There are many parallels between TB and COVID-19, the influence of misinformation and the failure of the world's major powers to collaborate resulted in a staggering death toll (148). Despite these failings, there are opportunities for learnings and the COVID-19 vaccine development response shows what can be achieved when there is political will and adequate resource allocation (149). Many of the technological advances and behavioral changes that were successful in tackling COVID-19, are of translatable value to combating TB e.g., technological developments in contact tracing, risk assessment and improved interconnected real time data, portable diagnostics, improved supply chain management, effective public health communication, and the application of non-pharmaceutical interventions (149–151). Increased resourcefulness is also required when tackling TB during conflict, which compounds the determinants of TB and the risk of drug resistance, whilst amplifying social inequities and vulnerabilities (152, 153). Conflict leads to the diversion of economic resources, food insecurity, patient delays in seeking care, healthcare disruption, and displacement (152, 154). During conflict, strong local leadership and community engagement are required as many stakeholders cannot actively work in the field due to security and logistical reasons (155, 156). Other innovations that have been implemented to address the additional complexities of conflict include self-administered therapy (SAT), emergency drug packs, use of technology to maintain communication and diagnostic investments (157–159). For innovations that are applied to mitigate climate change they must ensure that they do not exacerbate the inequities that climate change already creates (160). It is the populations who have contributed least to climate change that are the most vulnerable to its impact, and mitigation strategies that impact food and energy prices also have their greatest impact on these populations (160). Research shows climate change to be positively associated with a number of TB risk factors including poverty, undernutrition, HIV, diabetes and overcrowding (161). In addition to affecting environmental factors, climate change has been shown to impact TB epidemiology through its impact on both the host and the pathogen (162). Increasing temperatures are associated with increased bacterial growth and the collateral development of antibiotic resistance, and changes in ultra-violet radiation are associated with Vit D metabolism and BCG vaccine efficacy (162, 163).

In addition to the SDH, are a number of health and demographic factors that also need to be taken into consideration within the context of the TB pandemic. HIV infection is the greatest risk factor for LTBI progressing to active disease (15–22 times more likely) and TB is the major cause of HIV-related deaths (164, 165). Many of the SDH that increase vulnerability to TB, also increase vulnerability to HIV/AIDS, pandemic vulnerabilities that are amplified by COVID-19 and conflict (166–168). The COVID-19 pandemic highlighted the need to reorientate primary healthcare provision and the WHO now recommends an integrated rather than vertical approach (169), which has been shown to improve TB treatment outcomes and expediate ART initiation (170). Like HIV, diabetes has a synergistic role with TB, people with diabetes are at increased risk of developing TB and experiencing poor treatment outcomes, and TB and TB medications can impair glucose tolerance (171, 172). Diabetes is a growing health concern, estimated to impact 6.1% of the global population in 2021, and projected to affect 9.8% by 2050 (173). The disease has its greatest impact in low- and middle-income countries, which were estimated to carry 80% of the global burden in 2019 (174). The need to integrate TB and diabetes care is gaining increasing recognition (175, 176), and where co-management has been implemented, improved patient outcomes are being achieved (177). The most common form of diabetes is type-2 and like TB, its prevalence increases with age (178, 179). Many aspects of aging present challenges to TB control and these will become of increasing importance as the global demographic ages. By 2030, it is estimated that one in six people will be over 60 years of age and that by 2050, the population in this age group will have doubled (180). Aging is associated with immunosenescence, malnutrition, comorbidities, cognitive disorders, functional dependence, drug-drug interactions for those on polymedication and the possibility of poorer tolerance of TB drugs (179). Although multidisciplinary management is advocated for in geriatric health facilities (179, 181), these concepts are unlikely to be applicable in low-income settings where the elderly are cared for by family. Concepts, policies, and frameworks are also lacking to address post-TB sequelae across all patient populations (182). There is increasing evidence to show that TB has frequent and substantial morbidity consequences post microbiological cure (183), and there is a need to consider these sequelae in TB healthcare provision. Although some interventions have been shown to be effective at reducing the burden of post-TB sequelae, further research is urgently required as a significant population is impacted, with the number of TB survivors estimated to be alive in 2020 being more than 10 times the estimated annual disease incidence (182, 184, 185).

### TB funding

Addressing the TB epidemic will require, sustained, long-term, funding. The Global Plan to End TB, 2023–2030 (Global Plan) estimates that US$249.98 billion will be required over this time frame if TB is to be eradicated as a public health challenge by 2030 (186). The significantly higher funding needed in the current Global Plan, relative to the previous plan, is attributed to catching up on historic underfunding, mitigating the consequences of COVID-19 and fast tracking the development of new tools including the development of at least one new vaccine (186). However, significant under-funding continues to be a major barrier with political commitments not being upheld (187, 188). The World's response to the COVID-19 pandemic however, shows what can be achieved when there is significant political will and adequate resource allocation (186). The financing disparity between COVID-19 and TB reflects the global inequity in our approach to healthcare, despite TB modeling showing a 40:1 return on investment and the potential loss of US$1 trillion and 234 million DALYs between 2023–2030 if the status quo is maintained (186, 187).

The current levels of funding for TB research and development (R&D) are insufficient, with $1.03 billion invested in 2022 vs. an annual target of $2 billion as pledged by World leaders at the U.N. high level meeting on TB in 2018 (189). Over half of the 2022 TB R&D funding originated from two organizations- the United States National Institutes of Health and the Bill & Melinda Gates Foundation (189). In 2021, 79% of funding for TB prevention, diagnosis and treatment was derived from domestic sources with the split of funding (domestic vs. international) shown to be highly variable between countries (190). The Global Fund to fight HIV, TB, and malaria is a major source of funding for TB, with its contribution equating to 76% of international donations in 2021 (190). Proposed strategies to combat TB form the basis of funding applications to the Global Fund, which enables each country to tailor their response in consideration of their cultural, political, and epidemiological context (191).

### Patent barrier and monopolies

Although patents are necessary to incentivize innovation and R&D investment, they can be exploited for financial gain to the detriment of the people the innovation is intended to serve (192). A pertinent example is Johnson and Johnson (J&J) profiteering from bedaquiline, and Otsuka profiteering from delamanid, with treatment regimens estimated to cost 13–18 times and three times more than the cost of production plus profit, respectively (193, 194). Both pharmaceutical companies are also exploiting their monopoly positions by enforcing secondary patents and utilizing opaque restrictive licenses that undermine competition (193, 194).

Similar corporate extortion, to the detriment of those most in need, occurs with TB diagnostic tools (195). Although Cepheid has made marginal reductions to the cost of its GeneXpert technology (196), prices remain at more than triple to cost of production and petitions continue to seek further price reductions (195, 197). The extent of corporate greed is exacerbated by the fact that significant public funding has supported the development of these technologies; Cepheid received $250 million to develop GeneXpert (195), and direct and indirect public contributions to the development of bedequiline are estimated at $455–747 million (198).

## End TB strategy pillar III: intensified research and innovation

### Artificial intelligence

Artificial Intelligence (AI) is the simulation of human cognitive function using computer systems (199). AI techniques include machine learning that employs multiple algorithms to identify complex nonlinear relationships within large datasets, deep learning where multiple layers of processing are used to progressively extract higher level features, and generative AI that can create new content (199, 200). AI has revolutionized healthcare and can be applied to improve disease screening and diagnosis, optimize treatment regimens and advance precision medicine, support drug and vaccine development, optimize health systems management, support disease surveillance and refine disease risk predictions, assist clinical care and improve patient education (201, 202). AI tools can also improve accuracy and efficiency while reducing costs and providing a solution to the universal shortage of healthcare professionals (201, 203).

Although AI presents many opportunities, ethical, privacy, data security, algorithm biases, and legal considerations present complex challenges and further clinical trials are required to verify the relevance of AI models (201, 204). AI algorithm development is reliant upon large well annotated datasets which is challenged by the complexities of accessing sensitive personal data (205). The challenges of accessing sensitive personal data include the technological implications of data protection and definitions of confidentiality and other core tenets of medical ethics (206). AI algorithms may reflect human biases in decision making and the biases that are inherent in current healthcare delivery (206). The legal complexities regarding the application of this technology include the lack of universal definitions and guidelines for its use, and overlapping theories of liability across multiple stakeholders (207, 208). It is important to ensure that the business models used to develop, deploy and support these technologies do not embody typical political power relations that reproduce past inequities (209).

Despite these complexities, AI is providing a transformative role in TB control. In 2022, the WHO launched an operational research package to generate data on treatment decision algorithms for pulmonary TB in children (210). This operational research seeks to provide external validation of two decision algorithms designed to help clinicians determine whether to commence treatment (210). Computer-aided detection (CAD) software is being utilized to automate the interpretation of digital X-ray images and is now recommended as an alternative to human interpretation in WHO screening guidelines (211). WHO recommend the use of CAD for screening and triage when interpreting plain X-ray s from patients ≥15 years old (211). CAD has the potential to improve access to screening as it can operate in both portable and ultra-portable X-ray machines, but it has the disadvantage of requiring calibration with local data due to heterogeneity in the accuracy of threshold scores and can only be applied to frontal X-ray images for adults (212). AI provides an opportunity to increase the efficacy of resources which in many high TB burden countries are constrained. The application of this technology to patients on directly observed therapy (DOT) has been successful in reducing the time and cost burden on both patients and healthcare workers (213, 214). The application of AI to monitor treatment efficacy and predict prognosis is being explored (215). AI is successfully predicting treatment duration, adverse reactions, drug resistance, and treatment outcomes which paves the way for personalized care and improved treatment outcomes (204, 216).

### Evolving technologies and innovative interventions/applications

Advances in geographic information systems (GIS) have played a pivotal role in analyzing and visualizing complex health data to inform evidence-based decision making (217). GIS applications provide an opportunity to evaluate the intricacies between disease and environmental, socio-economic and demographic factors (218). Although there are challenges (e.g., data quality, data privacy, and resource constraints) emerging technologies and improved spatial resolution hold promise for advances in this field (218).

Digital health technologies have a number of innovative approaches that the WHO recommend in support of the End TB Strategy (219). Technologies to improve patient engagement and treatment adherence include telemedicine, electronic medication monitors, video supported treatment and mobile phone messaging (219). Electronic data systems are of increasing importance for patient care, TB program management and surveillance systems (219). Online technologies are an effective education tool and have potential to support mental health interventions (220, 221).

Drones have been used to provide cost-effective bi-directional transport of sputum samples and TB medications between remote communities and diagnostic/treatment facilities (222). Drones provide an opportunity to support TB control programs in resource limited settings that often have limited laboratory and transportation infrastructure, limited healthcare system coverage, a paucity of healthcare professionals and the challenge remoteness and vulnerability to natural disasters (222, 223).

The use of renewable energy as facilitated access to modern medical technologies for populations without access to electricity. The installation of solar photovoltaic systems within health systems as part of the United Nations Development Programme (UNDP) Solar for Health is proving to be fundamental to the success of many TB programs (224–226). Advances in battery technology have also made significant contributions to the feasibility of utilizing renewable energy solutions in healthcare (227). Battery solutions in conjunction with the development of mobile technologies e.g. portable X-rays, enable remote communities to access TB healthcare (228, 229).

Nano medicine has a number of potential applications including mycobacterial strain identification and efficient medication delivery (230). Nanoparticles are being evaluated for their ability to diagnose both latent and active infection and discriminate specific mutations associated with antibiotic resistance (230, 231). Nanomedicine has the potential to deliver targeted, long-lasting antibiotics within the therapeutic range, thereby avoiding the toxicity and non-compliance issues arising from conventional therapy (232).

An alternative approach to conventional therapies is Host-Directed Therapy (HDT), which uses agents to modulate host immunity and enhance the efficacy of anti-tubercular drugs (233). Potential HDT agents include antimicrobial peptides, micronutrients, and re-purposed drugs but further clinical trials are required to evaluate their safety and efficacy alone and in combination with anti-TB drugs (233).

### Mycobacteriophages

The increasing health security threat of antibiotic resistance, and advances in molecular biology, have led to a resurgent interest in the therapeutic use of bacteriophages (phages) (234). Phages are viruses that infect bacteria and kill their host cells in the lytic replication phase of their lifecycle (235, 236). Phages are host specific, with the specificity of some limited to strains within a bacteria species (237).

Mycobacteriophages are phages of the genus *Mycobacterium* and were first isolated in 1954 (236). Although phages are ubiquitous and considered to be one of the most abundant biological agents on earth, the slow growth rate of MTB creates technical challenges to their isolation (237, 238), and to date the efficacy of phages against MTB infection in humans has not been evaluated (239), Conversely, the fast-growing non-pathogenic mycobacterium *M. smegmatis*, has provided an ideal host platform from which >10,000 phages have been isolated and of these >2,000 have been sequenced (238). Despite the significant number of isolates, there is very limited data on their potential role in the treatment of infection (240), and phages have only been used on compassionate grounds for patients with highly antibiotic-refractory NTM infections (235). While these NTM cases were limited in number, and outcomes confounded by complex clinical conditions, it is hoped that they can inform future trails (238).

Future trials will need to evaluate phage efficacy, further elucidate their strengths and limitations and determine optimal doses, methods of administration, potential antibiotic interactions and the impact of antibiotic resistant hosts (235, 239). Phages have the advantage of high host specificity, the capacity to replicate and although data is limited, reported safety profiles are high and adverse reactions rare (235). High host specificity can however be a double edged sword, by limiting the development of generalized treatments and necessitating the need for patient specific treatments (235). Phages also have the disadvantage of being potential targets of the human immune system, and their large size relative to antimicrobial drugs, can impact how and where they can travel in the body (235, 237). Although phages can access extracellular bacteria, their ability to infect intracellular bacteria or bacteria within granulomas may be limited (235). In mice, phage aerosols have been shown to reduce MTB burdens prior to macrophage uptake and granuloma formation, leading to the suggestion that they may also be utilized as a prophylactic agent to minimize transmission in high-risk settings (241). Although phages have mechanisms to evade bacteria host defense mechanisms and selection pressure favors their fitness, the same selection pressures apply to the host cells, making phage resistance a future consideration (238). Combined phage-antibiotic therapies present a synergistic challenge to the bacteria host cell that maybe beneficial to combating infection (242). To counter the development of phage resistance when used as the sole therapeutic agent, phage cocktails or sequential treatments have been proposed (242).

## Conclusions

Although TB continues to elude eradication, many new technologies and applied applications offer exciting prospects in working toward the End TB Strategy targets. Although these new technologies offer promise, one of the greatest challenges will be ensuring that those in need have equitable access. Addressing underlying inequities, which also result in the social determinants of the disease, will be one of the biggest challenges to overcoming the TB pandemic.
